# Case Report: The leiomyoma-leiomyosarcoma sequence exists in the esophagus: first case of malignant transformation treated with endoscopic submucosal dissection

**DOI:** 10.3389/fmed.2025.1716368

**Published:** 2026-01-12

**Authors:** Xu Wang, Gen Gui, Ruixue Liu, Zhaoyun Yang, Guifang Xu, Bin Sun

**Affiliations:** 1 Department of Gastroenterology, The First Affiliated Hospital of Anhui Medical University, Hefei, Anhui, China; 2 Anhui Provincial Key Laboratory of Digestive Disease, The First Affiliated Hospital of Anhui Medical University, Hefei, Anhui, China

**Keywords:** endoscopic submucosal dissection (ESD), esophageal leiomyoma, leiomyoma-leiomyosarcoma sequence, leiomyosarcoma, malignant transformation

## Abstract

**Objectives:**

Esophageal leiomyosarcoma (ELMS) is an extremely rare malignant tumor, and the possibility of malignant transformation from benign esophageal leiomyoma has historically been dismissed. This report presents, to our knowledge, the first documented case of malignant transformation within an esophageal leiomyoma and its successful treatment via endoscopic submucosal dissection (ESD), challenging the conventional belief regarding the absolute benignity of esophageal leiomyomas.

**Case summary:**

A 52-year-old female presented with non-specific symptoms and was found to have a small (0.8 cm) esophageal submucosal nodule during gastroscopy. Endoscopic ultrasound (EUS) showed a well-defined hypoechoic lesion originating from the submucosal layer. The lesion was completely resected via ESD. Histopathological examination demonstrated a conventional leiomyoma with a focal area of malignant transformation to leiomyosarcoma, characterized by high cellularity, nuclear atypia, increased mitotic activity [5 per 2 high-power fields (HPF)], elevated Ki-67 index (40%), and loss of smooth muscle markers (SMA, desmin) in the malignant region. Surgical margins were tumor-free. The patient recovered well with no recurrence at 60-day follow-up.

## Highlights

- We report what we believe to be the first documented case of malignant transformation within an esophageal leiomyoma, supporting a “leiomyoma-leiomyosarcoma sequence” in the esophagus.
- Successful complete resection achieved via endoscopic submucosal dissection (ESD), offering a minimally invasive treatment option.
- Histopathology revealed a focal sarcomatous transformation with high Ki-67 index (40%) and loss of smooth muscle markers (SMA, desmin).
- Challenges the traditional dogma that esophageal leiomyomas are invariably benign and have negligible malignant potential.
- Highlights the importance of advanced endoscopic evaluation and meticulous pathological examination for seemingly benign submucosal tumors.
- Suggests that ESD may be a viable curative option for early-stage malignant transformations with negative margins.

## Introduction

Esophageal leiomyosarcoma (ELMS) is an exceptionally rare malignant mesenchymal tumor, accounting for less than 1% of all esophageal malignancies (1, 2). It originates from the smooth muscle layers of the esophageal wall and is distinguished from the more common gastrointestinal stromal tumors (GISTs) by its unique immunohistochemical profile, typically demonstrating positivity for smooth muscle markers (e.g., SMA, desmin) and negativity for CD117 and DOG-1 (3). Clinically, ELMS often presents with non-specific symptoms such as progressive dysphagia, retrosternal pain, or weight loss, typically in middle-aged and elderly patients (4, 5). Owing to its rarity, most evidence regarding ELMS is derived from case reports or small series, complicating the establishment of standardized diagnostic and therapeutic guidelines.

The biological behavior of ELMS is characterized by slow growth and a propensity for late metastasis, often resulting in a more favorable prognosis than esophageal squamous cell carcinoma or adenocarcinoma, provided complete surgical resection is achieved (6, 7). The cornerstone of treatment is radical surgical excision, traditionally performed via open approaches, though minimally invasive techniques such as thoracoscopic and laparoscopic esophagectomy are increasingly reported (8, 9). The role of adjuvant radiotherapy or chemotherapy remains controversial and is generally reserved for unresectable or metastatic disease (10).

A particularly enigmatic aspect in the oncogenesis of smooth muscle tumors is the potential for malignant transformation from a pre-existing benign leiomyoma. While a well-documented “leiomyoma-leiomyosarcoma sequence” exists in the uterus (11), this phenomenon has been historically considered virtually non-existent in the gastrointestinal tract, particularly the esophagus. Conventional teaching holds that esophageal leiomyomas possess an exceedingly low malignant potential (<0.1%) and are effectively cured by local excision, often via endoscopic submucosal dissection (ESD) (12). A comprehensive literature search of PubMed/MEDLINE databases was conducted up to December 2025 using search terms including “esophageal leiomyosarcoma,” “leiomyoma malignant transformation,” and “gastrointestinal stromal tumor.” While primary esophageal leiomyosarcomas have been reported, and endoscopic techniques have been applied to gastric mesenchymal tumors (13), no previous report described histopathological evidence of malignant transformation arising within a pre-existing esophageal leiomyoma. However, this long-standing dogma is being challenged. A seminal report by Yamamoto et al. described the first incontrovertible case of gastric leiomyosarcoma arising from a leiomyoma, demonstrating a clear transition zone with divergent immunohistochemical features (e.g., elevated Ki-67, p53 overexpression, and loss of smooth muscle markers in the sarcomatous component) (14), suggesting a potential shared oncogenic pathway across anatomical sites.

Herein, we present a groundbreaking case of a 52-year-old female with a small esophageal submucosal tumor that was completely resected via ESD. Histopathological examination revealed a previously unreported finding: a conventional esophageal leiomyoma with a focal area of malignant transformation to leiomyosarcoma. This case represents, to our knowledge, the first documented instance of an esophageal leiomyoma-leiomyosarcoma sequence treated with ESD. We integrate this case with a systematic review of the recent literature on primary ELMS, highlighting the clinical, pathological, and therapeutic nuances of this rare entity and challenging the traditional belief regarding the malignant potential of esophageal leiomyomas.

## Case presentation

A 52-year-old female presented to a local hospital with complaints of activity-induced chest tightness, dyspnea, and limb numbness. Gastroscopy performed on 21 April 2025, revealed an esophageal submucosal protrusion: a 0.8 cm × 0.8 cm submucosal nodule located 25 cm from the incisors. Physical examination upon admission showed the patient was in generally good condition. No significant cardiopulmonary abnormalities were detected. The abdomen was soft, and non-tender without hepatosplenomegaly. Her medical history included hypertension (>4 months) managed with daily amlodipine 5 mg, laryngeal polyps (>10 years) with prior unspecified treatment, lumbar disk herniation (>1 year), and tubal ligation (1993).

**FIGURE 1:**
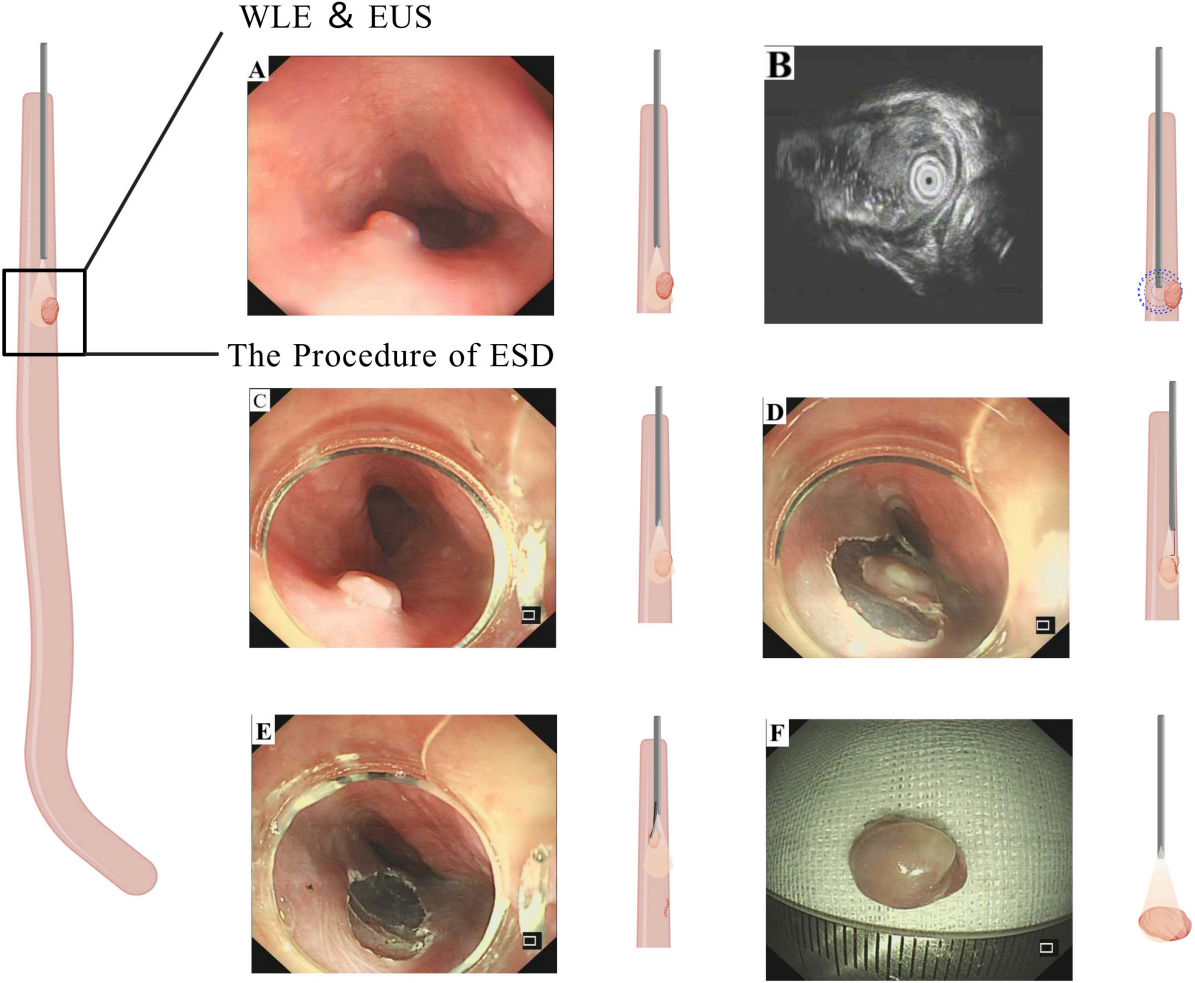
Preoperative endoscopic, endoscopic ultrasound (EUS) evaluation of the esophageal submucosal lesion and procedural steps of endoscopic submucosal dissection (ESD). **(A)** White light endoscopic view demostrates a smooth, rounded submucosal nodule (arrow) located 22 cm from the incisors. The overlying mucosa is intact without evidence of ulceration or erosion. **(B)** Corresponding EUS image reveals a well-defined, hypoechoic mass (calipers) arising from the submucosal layer (layer 3). The lesion exhibits relatively heterogeneous internal echogenicity. The muscularis propria (layer 4) remains intact deep to the lesion. **(C)** White-light endoscopic view confirming the submucosal nodule (arrow) prior to intervention. **(D)** Circumferential mucosal incision around the lesion using a dual knife after successful submucosal lifting. **(E)** Post-resection mucosal defect (ulcer bed) following complete en bloc removal. The underlying muscularis propria is visible and appears intact. **(F)** Macroscopic photograph of the resected ESD specimen *ex vivo*. The specimen consists of a single, oval-shaped tissue fragment. A ruler is included for scale, confirming the dimensions (∼1.0 cm × 0.8 cm).

On 28 April 2025, endoscopic ultrasonography (EUS) identified a solitary submucosal nodule in the esophagus at 22 cm from the incisors. The overlying mucosa appeared smooth without surface erosion or ulceration. EUS demonstrated a well-defined, hypoechoic mass within the submucosal layer (third layer), measuring approximately 5.4 mm × 4.3 mm, with heterogeneous internal echogenicity and intraluminal protrusion. The underlying muscularis propria (fourth layer) remained intact. The preliminary diagnosis was an esophageal submucosal lesion, likely leiomyoma (Figure 1).

Endoscopic submucosal dissection (ESD) was performed on 30 April 2025, under general endotracheal anesthesia with CO_2_ insufflation. White light endoscopy confirmed the nodule at 22 cm. Procedural details: An AQ260J endoscope with distal cap was employed. Submucosal injection of a solution containing sodium hyaluronate, normal saline, adrenaline, and indigo carmine achieved adequate lifting. Circumferential incision was made with a dual knife, followed by meticulous submucosal dissection to accomplish en bloc resection. Hemostasis was secured with hot forceps. The specimen (1.0 cm × 0.8 cm) was retrieved for pathological analysis (Figure 1). The procedure was uncomplicated, with minimal blood loss and stable hemodynamics.

Gross examination revealed a single grayish-white polypoid tissue fragment (0.8 cm × 0.5 cm × 0.4 cm) from the mid-lower esophagus. Microscopic analysis demonstrated two distinct histological patterns: (1) A peripheral leiomyoma-like area showing orderly fascicular architecture of spindle cells with minimal atypia and rare mitoses (Ki-67 index < 2%), with strong immunohistochemical positivity for smooth muscle markers; (2) A central sarcomatous transformation zone (0.4 cm × 0.2 cm × 0.2 cm) exhibiting high cellularity, marked nuclear pleomorphism, increased mitotic activity [5/2 high-power fields (HPF)], and elevated proliferation index (Ki-67 approximately 40%), with loss of smooth muscle immunomarkers.

**FIGURE 2:**
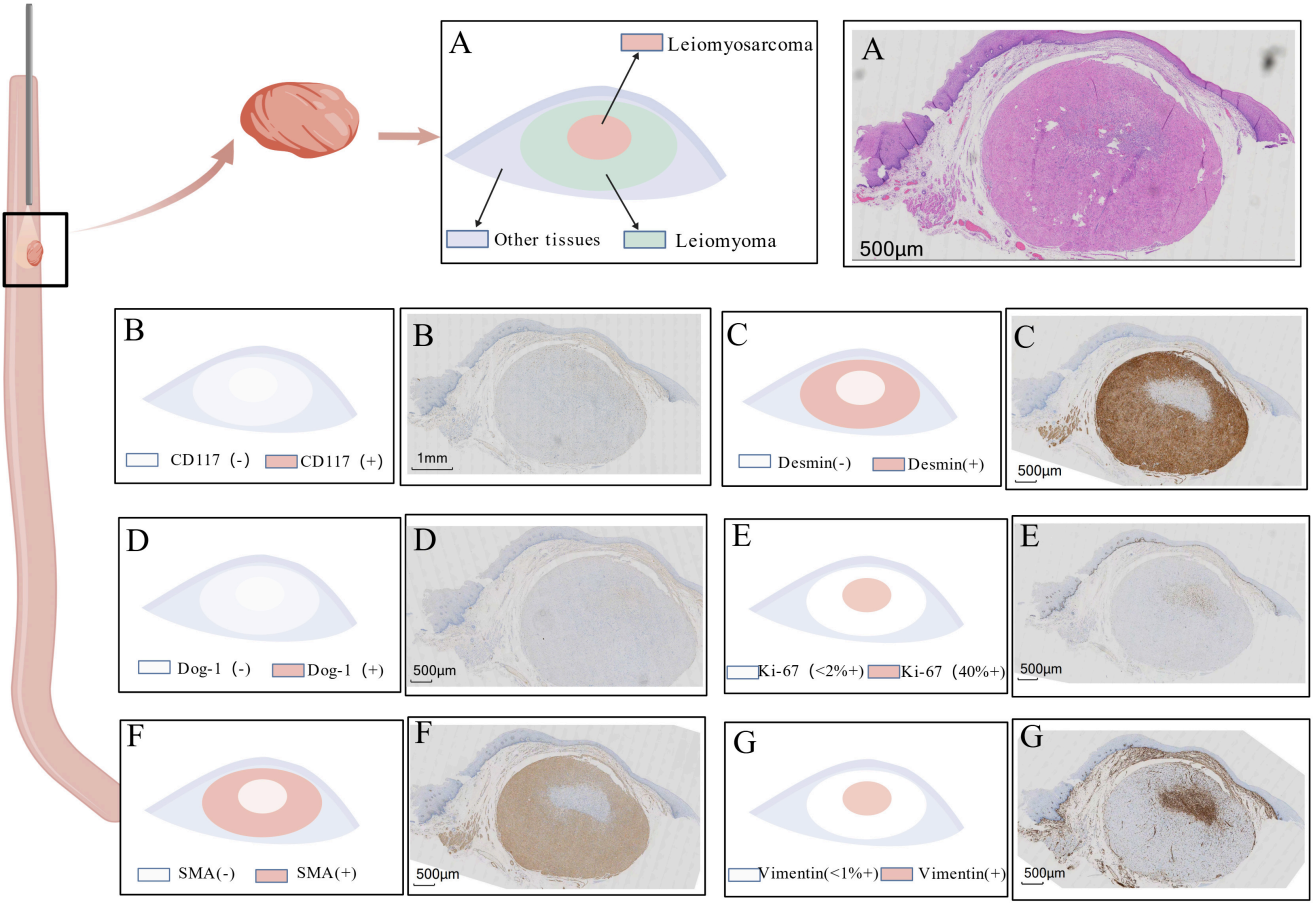
Histopathological and immunohistochemical characteristics of the resected tumor. **(A)** Hematoxylin and Eosin (H&E) stain: Overview demonstrating a transition zone (dashed line) between the peripheral leiomyoma-like area (left), composed of spindle cells arranged in orderly fascicles, and the central sarcomatous area (right),whch exhibits hypercellularity, marked nuclear atypia, and increased mitotic activity (inset, arrow indicates a mitotic figure). **(B–G)** Immunohistochemical staining results: **(B)** CD117: Negative in both tumor components, ruling out GIST. **(C)** Desmin: Strongly positive in the peripheral leiomyoma area (left) and completely negative in the central sarcomatous area (right). **(D)** DOG-1: Negative throughout the lesion, further supporting the exclusion of GIST. **(E)** Ki-67: Low proliferation index (<2%) in the leiomyoma area (left), contrasting with a high proliferative index (approximately 40%) in the sarcomatous focus area (right). **(F)** SMA (Smooth Muscle Actin): Strongly positive in the leiomyoma area (left) and markedly decreased/absent in the sarcomatous area (right), indicating loss of smooth muscle differentiation. **(G)** Vimentin: Diffusely positive in the sarcomatous area (right), consistent with a malignant mesenchymal phenotype; weak or focal positivity may be observed in the leiomyoma area.

The overlying mucosa was intact, excluding sarcomatoid carcinoma (Figure 2).

Immunohistochemical resultsf: (1). Leiomyoma area: Ki-67 (<2%+), SMA (Strongly+), Desmin (Strongly+), Vimentin (Focally weak+), CD117 (−), DOG-1 (−), ERG (−). (2). Sarcomatous area: Ki-67 (Approximately 40%+), SMA (−), Desmin (−), Vimentin (Diffusely+), CD117 (−), DOG-1 (−), ERG (−) (Figure 2).

Final diagnosis: Esophageal leiomyoma with focal malignant transformation (leiomyosarcoma), 0.8 cm × 0.4 cm × 0.2 cm, without lymphovascular or perineural invasion, and with clear resection margins.

Postoperative management included magnesium (20 mg BID) and thymoprotein oral solution (30 mg BID). No recurrence or etasmtasiswas observed at 60-day follow-up.

## Discussion

The present case delineates a paradigm-shifting occurrence: the malignant transformation of a histologically conventional, small (0.8 cm) esophageal leiomyoma into a leiomyosarcoma, successfully treated with ESD. This finding challenges the entrenched clinical axiom that esophageal leiomyomas possess negligible malignant potential and are invariably benign (12). Our case, demonstrating focal sarcomatous transformation with a high Ki-67 index (40%) and loss of smooth muscle actin (SMA) and desmin expression within a background of typical leiomyoma, provides compelling histopathological evidence for a “leiomyoma-leiomyosarcoma sequence” in the esophagus. This phenomenon mirrors similar transformation recently described in the stomach (14) and colon (15), and is well-established in uterine pathology (11).

The malignant transformation of benign smooth muscle tumors is predicated on the accumulation of genetic aberrations. In uterine leiomyosarcomas, molecular studies have evidenced that a significant subset arises from pre-existing leiomyomasthrough acquisition of additional driver mutations in pathways involving TP53, RB1, and MYC amplification (11). The immunohistochemical profile in our case strongly supports a similar mechanism. The dramatic upregulation of Ki-67 and complete loss of SMA and desmin expression in the malignant focus indicate not only rampant proliferation but also a dedifferentiation process. While TP53 dysfunction was not investigated in this case, its potential role warrants future investigation given that overexpression a common feature in transformed zones elsewhere (14). This case, therefore, suggests the need for a re-evaluation of esophageal smooth muscle tumor biology., suggesting that even small, endoscopically benign-appearing leiomyomas may harbor malignant potential and require a more nuanced diagnosis and management approach. It is important to note that confirmatory molecular analyses (e.g., for TP53, RB1 mutations) were not performed in our case. While the immunohistochemical profile strongly suggests a dedifferentiation process analogous to that seen in uterine tumors, the specific genetic drivers remain unconfirmed and warrant future investigation.

This discovery mandates critical re-appraisal of diagnostic strategies. In our case, standard white-light endoscopy and EUS revealed a typical small submucosal lesion with homogeneous echogenicity and clear borders, providing no preoperative indication of internal heterogeneity or malignant focus. This underscores the limitation of conventional imaging. Advanced endoscopic modalities could prove pivotal in identifying “at-risk” lesions preoperatively. Contrast-Enhanced EUS (CE-EUS) might reveal aberrant neovascularization or necrotic areas within the tumor (16), while endoscopic elastography could potentially detect foci of increased stiffness within a softer leiomyoma background, as sarcomatous tissue typically exhibits a higher Young’s modulus (17). We propose that any esophageal submucosal tumor (SMT) showing internal heterogeneity on EUS, regardless of size, should undergo deep targeted sampling. Although EUS-FNA demonstrates >90% diagnostic accuracy for gastric SMTs (18), its utility in esophageal leiomyosarcoma remains less documented. Given the challenges in obtaining diagnostic material from these tumors due to necrosis and overlying mucosa, EUS-FNA with rapid on-site evaluation (ROSE) could provide valuable diagnostic information before treatment planning (19). To facilitate the systematic integration of these advanced techniques into clinical practice, we propose a preliminary diagnostic algorithm for esophageal submucosal tumors (SMTs) with indeterminate or heterogeneous features: initial characterization with standard EUS, followed by contrast-enhanced EUS (CE-EUS) to evaluate intralesional vascularity patterns, and supplemented by elastography for quantitative stiffness mapping. Lesions exhibiting suspicious features (e.g., irregular hyper-enhancement, focal hard areas within a soft background) should be prioritized for diagnostic sampling via EUS-guided fine-needle aspiration (EUS-FNA) with rapid on-site evaluation (ROSE). Prospective studies are urgently needed to evaluate the diagnostic yield, accuracy, and cost-effectiveness of this multimodal imaging approach in detecting occult malignant foci within seemingly benign SMTs.

The therapeutic success of ESD in this case offers new perspectives for managing early-stage, localized malignant transformations. The established standard care for definitive leiomyosarcoma remains radical surgical resection with lymph node dissection (4, 8). However, for minute malignant foci within otherwise benign tumor that are completely excised with negative margins (R0), as in our case, ESD may represent a potentially curative, organ-preserving minimally invasive intervention. This approach aligns with growing reports of successful endoscopic resection of small, well-demarcated gastrointestinal leiomyosarcomas, including a recent case of gastric leiomyosarcoma managed with ESD (13), as well as earlier reports of polypoid esophageal leiomyosarcoma (20). The critical determinant remains meticulous histological examination of the entire resected specimen. Immediate perpendicular sectioning through the specimen center is essential to identify potential central heterogeneity. Pathological reporting must detail the malignant component size, mitotic count (per 2 mm^2^), necrosis presence, and margin status. The role of adjuvant therapy in such scenarios remains undefined. Given the favorable outcome in our patient with surgery alone and the lack of supporting data, adjuvant therapy cannot be routinely recommended but should be considered individually within a multidisciplinary tumor board setting, particularly for tumors exhibiting high-risk histological features such as a large malignant focus (>0.5 cm), elevated mitotic rate (>5/10 HPF), or presence of lymphovascular invasion. Given the microscopic nature and complete resection (R0) of the transformed focus in our case, adjuvant therapy was not indicated. However, management decisions in similar scenarios should be individualized through review by a multidisciplinary tumor board, considering both traditional sarcoma risk factors and patient-specific variables. The potential role of emerging targeted therapies in the adjuvant setting for such microscopic residual risk remains an open question warranting investigation in larger, collaborative studies.

**FIGURE 3:**
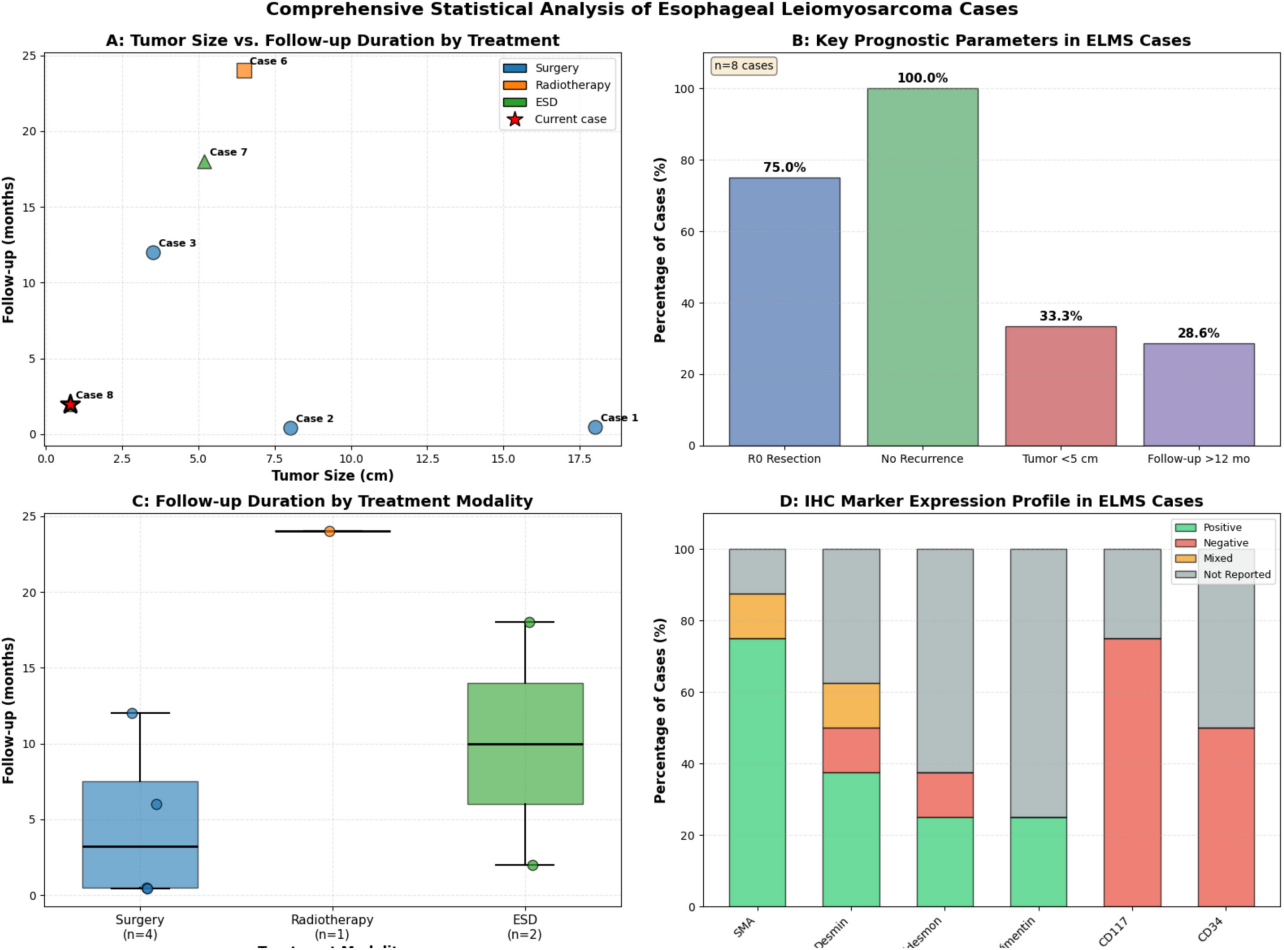
Comprehensive statistical analysis of primary esophageal leiomyosarcoma cases. **(A)** Scatter plot showing tumor size versus follow-up duration, with cases colored by treatment modality (Surgery: blue circles; Radiotherapy: orange squares; ESD: green triangles). The current case (malignant transformation) is highlighted with a red star. **(B)** Bar chart summarizing key prognostic parameters acrWang X, Gui G, Liu R, Yang Z, XuWang, Gui, Liu, Yang, Xu and Sun Bse series, including rates of R0 resection, recurrence-free status, small tumor size (<5 cm), and long-term follow-up (>12 months). **(C)** Box plot with individual data points showing follow-up duration distribution by treatment modality. **(D)** Stacked bar chart illustrating the expression profile of immunohistochemical markers in the reviewed ELMS cases, showing proportions of positive, negative, mixed, and not reported results for each marker.

To contextualize our findings, we conducted a systematic review of seven recent primary ELMS case reports (Table 1) (4, 8, 9, 19–22). The summarizes data reveal considerable diversity in presentation and management. Patients averaged 63 years of age with male predominance. Tumors were frequently large (average size ∼7 cm) and predominantly located in the middle and lower esophagus. Most cases underwent open esophagectomy, reflecting the advanced size and infiltrative nature at diagnosis. Our case remains unique in the series for its small size (0.8 cm), endoscopic respectability, and presentation as malignant transformation. Although follow-up data remain limited, they suggests that complete surgical resection, even for large tumors, can yield favorable long-term outcomes, consistent with the relatively indolent biology of ELMS compared to other esophageal cancers (4, 7). To facilitate comparative analysis of prognostic factors, we have synthesized key data from this case series in a new comprehensive figure (Figure 3). The visual summary highlights the unique position of our case (small size, endoscopic management) within the spectrum of reported ELMS and provides quantitative analysis of parameters including tumor size distribution, follow-up duration by treatment modality, and immunohistochemical expression patterns.

**TABLE 1:** Clinicopathological features of recently reported primary esophageal leiomyosarcoma cases.

case	References	Age	Sex	Gross morphology	Location	Size (cm)	Mitotic count (/10 HPF)*	Treatment	Follow-up	IHC profile	Outcome
1	Manipadam et al. (8)	68	M	Circumferential mural thickening, intraluminal polypoid	Mid-thoracic	18 × 9	35/10	Thoracoscopic esophagectomy + Gastric pull-up	14 days (Discharged)	SMA+, Caldesmon+	No recurrence at short-term FU; recommended XRT
2	Yusupbekov et al. (9)	58	M	Submucosal protruding lesion with ulceration	Upper third	8 × 8 × 8	N/R	McKeown esophagectomy	13 days (discharged)	N/R	Highly differentiated; no recurrence at 6 m
3	Ebi et al. (19)	82	M	Submucosal tumor	Lower esophagus	3.5 × 3.0	N/R	Surgical resection	12 months	α-SMA+, Caldesmon+, Desmin+; C-kit−, CD34−, S-100−	No recurrence at 1 year
4	Reddy et al. (4)	45	F	Large mass, ulcerated center communicating with lumen	Distal esophagus	N/R	2/10	Esophagogastrectomy	6 months	SMA+, Desmin+; CD34−, CD117−	Asymptomatic, no recurrence at 6 m
5	Reddy et al. (4)	52	M	Large heterogeneous mass, luminal narrowing	Subcarinal	N/R	4/10	SEMS Palliation + radiotherapy (45 Gy)	N/R	SMA+, Desmin+; CD34-, CD117-	Tolerating semisolid diet
6	Ma et al. (10)	48	F	Round soft tissue mass	Lower esophagus	6.5 × 4.0	N/R	Radiotherapy (IMRT, 60 Gy)	24 months	Vimentin+, SMA+; Cytokeratin−, EMA−, S-100−, C-kit−	Asymptomatic, no recurrence at 2y
7	Yamamoto et al. (20)	63	M	Polypoid tumor	Upper esophagus	2.5 ->5.2 (growth)	10/10	Endoscopic submucosal dissection (ESD)	18 months	SMA+, HHF35+; Desmin−, Caldesmon−, CD34−, c-kit−, S-100−	Disease-free at 1.5 years
8	our case	52	F	Submucosal (protrusion)	Esophagus (22 cm)	0.8	25 (equivalent in focus)*	Endoscopic submucosal dissection (ESD)	60 days	Leiomyoma area: SMA+, Desmin+, Ki-67 < 2% Sarcoma area: SMA−, Desmin−, Vimentin+, Ki-67∼40%	No recurrence at 2 m

*Mitotic counts are expressed per 10 high-power fields (HPF). For Case 8, the count in the sarcomatous focus was 5/2 HPF, equivalent to 25/10 HPF. M, Male; F, Female; N/R, not reported; HPF, high power field; IHC, immunohistochemistry; SMA, smooth muscle actin; SEMS, self-expanding metallic stent; IMRT, intensity-modulated radiation therapy; FU, follow-up; XRT, radiotherapy.

In conclusion, this report presents the first documented evidence of malignant transformation within an esophageal leiomyoma, successfully treated with ESD. This finding challenges the long-standing dogma of absolute esophageal leiomyomas benignity and introduces the concept of a leiomyoma-leiomyosarcoma sequence in the esophagus. These observations carry profound clinical implications: they highlight the need for a more vigilant diagnostic approach incorporating advanced endoscopic techniques for seemingly benign SMTs, emphasize the critical importance of thorough pathological specimen examination, and stimulate debate regarding potential role for minimally invasive therapy in very early-stage transformed lesions. Future investigations should focus on identifying predictive biomarkers and molecular signatures that could preoperatively stratify malignant transformation risk in esophageal leiomyomas.

## Limitations

This study has several limitations. First, it is a single-case report, which inherently limits the generalizability of the conclusions. Second, the follow-up period of 60 days is short for assessing the long-term recurrence risk of leiomyosarcoma, even for a small, completely resected (R0) focus. Continued and longer-term surveillance of this patient is imperative. Third, molecular profiling was not performed, leaving the genetic basis of the transformation speculative. Future multi-center studies and pooled analyses of similar rare cases are needed to validate our observations.

## Future perspectives

The hypothesis that small, endoscopically benign-appearing leiomyomas may harbor malignant potential, as suggested by this case, warrants rigorous validation through several approaches: (1) Establishment of multi-center registries to systematically collect and analyze similar rare cases; (2) Implementation of prospective molecular profiling of resected esophageal leiomyomas to identify driver mutations (e.g., in TP53, RB1, MED12) and epigenetic alterations associated with early transformation; and (3) Correlative studies linking preoperative imaging features (e.g., internal heterogeneity on EUS, specific elastography patterns) with detailed histopathological and molecular findings. The development of such integrated predictive models could ultimately guide risk stratification algorithms, determining which patients might benefit from more intensive surveillance or prophylactic resection.

## Conclusion

To our knowledge, this case provides novel and compelling evidence of a “leiomyoma-leiomyosarcoma sequence” in the esophagus, challenging the entrenched dogma of its absolute benignity. The successful, curative resection via ESD demonstrates a viable minimally invasive strategy for early-stage malignant transformations. These findings underscore the importance of heightened diagnostic vigilance for seemingly benign submucosal tumors and underscore the critical need for thorough histopathological assessment. Future research should focus on identifying predictive biomarkers to guide the management of esophageal leiomyomas.

## Data Availability

The datasets presented in this article are not readily available because of ethical and privacy restrictions. Requests to access the datasets should be directed to the corresponding authors.
